# Prevalence of intestinal parasitic infections among children in rural India

**DOI:** 10.6026/973206300214735

**Published:** 2025-12-15

**Authors:** Dipak Patanvadia, Pankti Pargi, Rekha Kishori, Amit Kumar, Deepak Deshkar

**Affiliations:** 1 Department of Microbiology, Zydus Medical College, Dahod, Gujarat, India

**Keywords:** IPIs (intestinal parasitic infections), stool examination

## Abstract

Intestinal parasite infections are triggered by climate, social status, occupation, personal hygiene, and educational status, resulting in malnutrition, diarrhea, blood loss, impaired work capacity, and decreased growth. Therefore, it is of interest to record Intestinal Parasitic Infections (IPIs) among youngsters. Hence, a total of 211 stool samples (115 male and 96 female) so as analyzed for macroscopic, microscopic, and occult blood. The analysis revealed that 43 out of 211 samples were positive. Thus, high prevalence of IPIs among children is observed. Hence, it is essential to limit parasitic infection by identifying the parasite at the species level.

## Background:

Intestinal parasite infections' is second most common contributor to morbidity and mortality of diarrhoeal Disease resulting from faecal contamination of food and water in development nations [1]. Amoebiasis, Giardiasis, Hookworm and Trichuriasis are the most common parasitic infection responsible malnutrition, diarrhoea, blood loss, impaired work capacity, reduce growth rate especially in children. There are several factors like educational status, occupational status, social economic status, poverty; social development process is significant contributor for increasing of intestinal parasitic infection that's why it is very important to control this sensitive issue both by socially and politically [2]. The prevalence of intestinal parasitic infection is varies according to age, sex, and geography [3]. According to world health organisation report 2020 approximately two billion people worldwide are affected by this infection, sub-Saharan Africa and South East Asia together accounting for more than 50% of the cases. In India 21% of India's population, may be afflicted by intestinal parasites of which About 39 million disability-adjusted life with intestinal parasitic infection [4]. It has been reported by many researchers that Prevalence of Intestinal parasitic infection was found to be about 1.2 times higher among children who did not consume albendazole (anti parasitic drug] than children who had taken albendazole. Some of the patients are asymptomatic that may act as a carrier can transmit infection to others specially in school children [5]. Globally, the most commonly reported parasitic infections include *Ascaris lumbricoides* (20%), Ancylostoma duodenale (18%), Trichuris trichiura (10%) and *Entamoeba histolytica* (10%) [6-7]. Good environmental sanitation and high standards of living could be major steps for reduction in the prevalence of intestinal parasites in the developed countries therefore it is of interest to report the prevalence of intestinal parasitic infection and the factors associated with it among age groups of 1 to 18 years [8]. Therefore, it is of interest to study of prevalence and determinants of intestinal parasitic infections among rural children in Dahod, Eastern Gujarat.

## Materials and Methods:

A cross-sectional study was conducted at Zydus Medical College and Hospital, Dahod, Gujarat, over a period of six months from 21 March 2023 to 21 September 2023 among schoolchildren aged 1-18 years. Individuals older than 18 years, those who were not permanent residents of the study area, and those receiving antiparasitic drugs were excluded from the study. The study was approved by the Institutional Ethics Committee of the institute.

## Sample collection:

Stool samples were collected in sterile, properly labelled containers and sent to the Department of Microbiology for routine examination.

## Macroscopic examination:

All stool samples were examined macroscopically for colour, consistency and the presence of mucus, blood, or adult parasites.

## Direct microscopy:

Each sample was examined microscopically using saline mount, iodine mount, and modified acid-fast staining techniques.

## Processing of samples:

The collected stool samples were processed for occult blood testing and examined using saline and iodine mounts, as well as modified acid-fast staining to detect the presence of occult blood, eggs, cysts/trophozoites and oocysts of *Cryptosporidium parvum*, respectively. In cases where the initial sample was negative, two consecutive stool samples were collected and examined.

## Analysis of data:

Data were entered in Microsoft excel, analysed by SPS software, plotted in the form of graph and pie chart and P value calculated by using chi square test.

## Results:

This investigation included 211 stool samples (115 males and 96 females), of which 43 were positive and 168 were negative for intestinal parasitic infections (IPIs) (Figure 1 - see PDF). Male patients (n = 30) were more frequently infected than female patients (n = 13). The most affected age group was 16-18 years (n = 17), with 11 infected males and 6 infected females. This was followed by the 11-15 years age group (n = 14), 6-10 years (n = 8), and 1-5 years (n = 4). In these age groups, the number of infected males and females was 9 and 5, 7 and 1, and 3 and 1, respectively (Table 1 - see PDF). *Giardia lamblia* (n = 21) was the most commonly detected parasite, followed by *Ascaris lumbricoides* (n = 8), *Hymenolepis nana* (n = 8), hookworm (n = 4), *Entamoeba histolytica* (n = 1), and *Strongyloides stercoralis* (n = 1). A higher number of *Giardia lamblia* cases (n = 6) were observed in males aged 11-15 years. *Hymenolepis nana* infections (n = 3) were equally distributed among males and females in the 11-15 and 16-18 age groups. Hookworm infection (n = 2) was observed in females aged 16-18 years, while *Entamoeba histolytica* (n = 1) was detected in a male aged 6-10 years, and *Strongyloides stercoralis* (n = 1) in a male aged 16-18 years (Table 2 - see PDF; Figures 2, 4, 5, 6, and 7 - see PDF). Among the 43 positive samples, two showed dual parasitic infections: hookworm with *Hymenolepis nana* and *Giardia lamblia* with *Entamoeba histolytica* (Figure 3 - see PDF and Table 3 - see PDF). Of the 43 infected cases, 31 (72.09%) were asymptomatic, while 12 (27.90%) were symptomatic (Table 4 - see PDF). None of the samples tested positive for occult blood or modified acid-fast staining.The present study evaluated eight variables to determine their association with IPIs: source of drinking water, treatment of drinking water, sharing food with friends, consumption of raw vegetables or fruits, nail trimming, family monthly income, habit of walking barefoot, and hand-washing practices. Among these variables, nail trimming (p < 0.00001), family monthly income (p = 0.015309), and hand-washing practices (p = 0.046605) showed statistically significant associations with IPIs, while the remaining variables were not significant (Table 5 - see PDF).

## Discussion:

Intestinal parasitic infections (IPIs) remain a major public health problem among children in developing countries such as India. In the present study, the prevalence of intestinal parasitic infections was higher in males (14.21% overall) than in females (6.16% overall). Similar findings were reported by Kaur *et al.* and Saurabh *et al.* who observed a predominance of infection in males (69.2%) compared with females (30.7%) and in males (59.4%) compared with females (40.5%), respectively. However, Patel *et al.* reported a higher prevalence among females (6.09%) than males (5.16%) [9, 10-11]. Several factors, including variations in sanitation and hygiene practices, public health infrastructure, food and water safety, nutritional status, host immunity, access to safe drinking water, socioeconomic conditions, and environmental determinants, may account for these differences in prevalence. In the current study, the most commonly affected age group was 16-18 years, with a prevalence of 8.05% (17/211 x 100). Several studies from India have reported a similar prevalence in the same age group [12, 13, 14-15]. The findings of the present study are in concordance with those of Tenali *et al.* from India, who reported a higher prevalence of intestinal parasitic infections among children aged 8-11 years [18]. Another study conducted by Singh *et al.* reported a higher incidence of IPIs in the 12-18-year age group [18-19]. The most common parasite identified in the present study was *Giardia lamblia* (43.75%), followed by *Hymenolepis nana* (18.6%). A study from Gujarat reported similar findings, with predominance of *Giardia lamblia* (28.99%) followed by *Hymenolepis nana* (20.29%). In contrast, Bajpai *et al.* reported *Entamoeba histolytica* (37.57%) as the most common parasite, followed by *Giardia lamblia* (23.12%) [16]. These variations in parasite prevalence may be attributed to differences in study populations, geographical regions, seasonal variations, and ethnic groups. In the present study, Giardia lamblia was most frequently reported in the 11-15-year age group, with a higher prevalence among males than females. However, many studies have reported a higher prevalence of Giardia lamblia in children younger than 10 years [17]. This difference may be due to the smaller number of samples from children under 10 years of age in the present study. Among the risk factors evaluated, nail trimming, family monthly income, and hand-washing practices showed a statistically significant association with intestinal parasitic infections. Similar findings were reported by Tenali *et al.* from India, who also observed significant associations between intestinal parasitic infections and nail trimming, family monthly income, and hand-washing practices [18]. In contrast, Adugna *et al.* from Ethiopia reported no significant association between these risk factors and intestinal parasitic infections, possibly due to differences in climate, socioeconomic conditions, and environmental factors [20]. Knani conducted a study in South Gujarat and reported a low prevalence of intestinal parasitic infections [21]. In the present study, hand washing and nail trimming were significantly associated with intestinal parasitic infections, whereas other risk factors were not significant. These findings are consistent with those reported by Ayubi *et al.* [22].

## Limitation of the study:

Present study was conducted on small sample size and did not provide detail about seasonal variations of parasite that will be remembered for further study.

## Conclusion:

Data shows the prevalence of intestinal parasitic infections is high. Therefore, it is essential to control these infections by identifying parasites at the species level to provide timely and appropriate treatment, along with improving personal hygiene, public sanitation, and the socioeconomic status of patients at both the individual and community levels.

## Competing interest:

The authors declared that they have no competing interest.

## Funding:

No funding

## Author's contributions:

The corresponding author contributed to writing of entire paper followed by first, second and third authors contributed to data collection and designing of the study.

## Ethical approval:

The study was approved by institutional ethical committee with letter number ZMCH/IEC150-2023/20- Microbiology. Consent from guardian of all participants was taken before sample collection and confidentiality of all participant maintained throughout the study.
